# Beyond a simple oxidation reaction: the complex molecular network regulating fruit and vegetable browning

**DOI:** 10.1093/hr/uhag060

**Published:** 2026-03-05

**Authors:** Jia Guo, Xinyi Duan, He Xu, Zishan Xu, Alisdair R Fernie, Yanjie Zhang

**Affiliations:** School of Life Sciences, Zhengzhou University, Zhengzhou, Henan 450000, China; School of Life Sciences, Zhengzhou University, Zhengzhou, Henan 450000, China; School of Life Sciences, Zhengzhou University, Zhengzhou, Henan 450000, China; School of Life Sciences, Zhengzhou University, Zhengzhou, Henan 450000, China; Max-Planck-Institut für Molekulare Pflanzenphysiologie, Am Mühlenberg 1, 14476 Potsdam-Golm, Germany; School of Life Sciences, Zhengzhou University, Zhengzhou, Henan 450000, China; Max-Planck-Institut für Molekulare Pflanzenphysiologie, Am Mühlenberg 1, 14476 Potsdam-Golm, Germany; Zhengzhou Research Base, State Key Laboratory of Cotton Bio-breeding and Integrated Utilization, School of Agriculture and Biomanufacturing, Zhengzhou University, Zhengzhou, Henan 450000, China

## Abstract

Fruit and vegetable browning is a complex physiological phenomenon responsible for substantial postharvest losses and profound economic consequences. While enzymatic oxidation mediated by oxidative enzymes has long been considered the core mechanism, emerging evidence highlights the flavonoid pathway as an alternative route, influencing pigmentation outcomes. Browning is governed by a multitiered regulatory network spanning molecular, biochemical, cellular, and physiological levels, which encompasses transcriptional, post-transcriptional, epigenetic, and hormonal controls. Notably, regulatory mechanisms exhibit both conserved features and species-specific variations, reflecting potential adaptive evolution that may underlie differential browning responses across species. Here, we provide a thorough review of current advances in the mechanistic understanding of browning, with emphasis on providing evidence on multilevel regulations, identifying conserved mechanisms versus species-specific variations, exploring their contributions to differential browning responses, and providing viable strategies for browning management through the application of exogenous hormones. Based on these, the current research landscape is critically assessed, and future research priorities are identified.

## Introduction

Fruit and vegetable browning is a common physiological phenomenon with major agronomic impacts. It is characterized by the production of dark/brown discoloration on the surface or inside the produce, and it contributes significantly to postharvest losses, being estimated to account for up to 50% of fruit losses in tropical regions [1]. Additionally, browning reduces the visual appeal and nutritional quality of fruits and vegetables, directly influencing consumers’ purchasing decisions and leading to significant market downgrading or outright rejection. Consequently, these factors translate into substantial economic losses across the supply chain [2]. As such, browning control extends beyond cosmetic considerations; it is crucial for minimizing food waste, enhancing resource efficiency, and ensuring agricultural economic sustainability.

Enzymatic oxidation represents the core mechanism of fruit and vegetable browning, through which oxidative enzymes convert phenolic compounds into melanogenic pigments. However, mounting evidence reveals that browning is more than a simple ‘enzyme–phenolic–oxygen’ reaction, and that alternative mechanisms are also involved [3, 4]. The browning phenotype additionally arises from the interplay between biological factors (e.g. the activity of browning-related enzymes, phenolic profiles, cellular energy status, membrane stability, and antioxidant capacity) and environmental cues (e.g. mechanical damage, water loss, pathogen infection, and chilling injury) [1, 2, 5]. This integrated process is finely modulated by a multitiered regulatory network spanning physiological, cellular, biochemical, and molecular levels. Furthermore, the composition of the network and mechanisms behind its regulation vary interspecifically, which contribute significantly to its complexity.

Core functional modules within this network include transcription factor (TF)-mediated gene regulation, microRNA-guided post-transcriptional regulation, epigenetic modifications, as well as hormonal signaling and responses. Rather than operating in isolation, these modules interact synergistically or antagonistically to fine-tune the browning process in plants. Therefore, a thorough understanding of this sophisticated molecular mechanism is crucial for devising effective control strategies, which remains a high priority in postharvest management. Substantial research efforts have been directed toward elucidating both the individual mechanisms and their integrative coordination within the regulatory system. Our current review synthesizes recent advances in understanding how these dynamically linked modules work individually and collectively to regulate browning and identifies both conserved mechanisms and interspecific variations across species.

## Enzymes involved in fruit and vegetable browning

**Figure 1:**
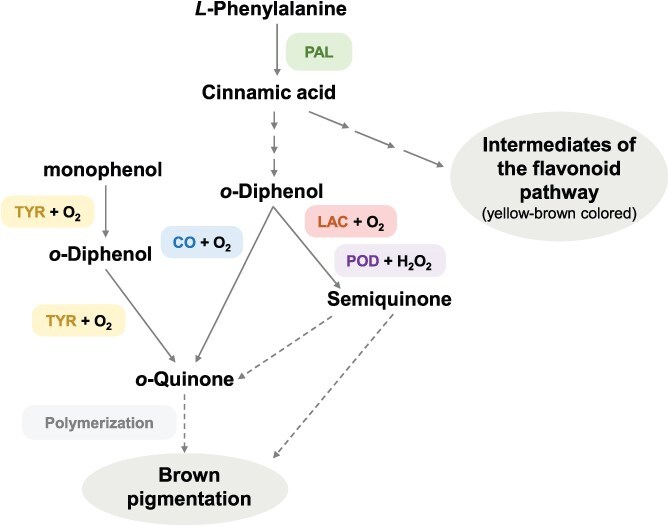
Schematic overview of pathways and key enzymes of enzymatic browning in plants. Solid arrows denote enzymatic reactions. Dashed arrows indicate reactions that do not require enzymatic catalysis. A series of arrows indicates multiple reactions catalyzed by distinct enzymes; However, the number of arrows does not correspond to the exact number of reactions. CO, catechol oxidases; LAC, laccase; PAL, phenylalanine ammonia lyase; POD, peroxidase; TYR, tyrosinase.

Fruit and vegetable browning, primarily caused by phenolic oxidation, is catalyzed by various classes of oxidative enzymes, including polyphenol oxidases (PPOs), laccases (LACs), and peroxidases (PODs) (Fig. 1; Table S1). Based on their reaction mechanisms and substrate specificities, PPOs can be further subclassified into two groups, namely catechol oxidases (COs) and tyrosinases (TYRs). In addition to phenolic oxidation, the accumulation of flavonoids or their precursors with yellow-brown color is also thought to contribute to browning in some fruit species [3, 4], and therefore, the involved biosynthetic enzymes, including phenylalanine ammonia lyase (PAL), also play indispensable roles in melanogenesis (Fig. 1).

### Catechol oxidase

Catechol oxidases (COs, EC 1.10.3.1) represent the most predominant type of PPOs (copper-containing metalloenzymes) in vascular plants [6] and the principal enzymes responsible for enzymatic browning in mechanically injured or senescing plant tissues [7, 8]. Mechanistically, COs catalyze the oxidation of *o*-diphenols into highly reactive *o*-quinones using molecular oxygen, which subsequently undergo non-enzymatic polymerization, forming insoluble brown pigments (Fig. 1). The critical role of CO-type PPOs in browning is supported by multiple lines of evidence: a strong correlation between PPO activity and browning severity was observed in potato [9]; the addition of PPO inhibitor or activator led to browning modulation in walnut (*Juglans regia*) [10]. Additional evidence includes the upregulation of three *PPO* genes (*CcPPO6/7/11*) in cut artichoke (*Cynara cardunculus* var. scolymus L.) capitula and their significant overexpression in brown-phenotyped calli [11]. Furthermore, targeted suppression of PPOs through CRISPR/Cas9-mediated gene editing in apples (10 *PPO*s) eggplant (*SmelPPO4*, *5* & *6*), potato (*StPPO2*), litchi (*LcPPO2*), and wheat (*TaPPO1*, *2*), as well as RNAi in apple, consistently leads to browning alleviation [7, 12–14], confirming the pivotal function of PPOs in enzymatic browning.

PPOs are mainly compartmentalized in plastids, while their phenolic substrates reside primarily in vacuoles. This spatial separation confines browning initiation to instances when cellular integrity is compromised (e.g. on cutting or bruising). Cellular damage triggers interconnected physiological disruptions that promote PPO-mediated browning [11, 15]. As observed in eggplant, the increased membrane permeability caused by lipoxygenase/phospholipase D-mediated lipid peroxidation facilitates PPO–phenolic contact, causing browning [15]. The accumulation of reactive oxygen species (ROS), resulting from mechanical stresses, accelerated membrane damage and elevated Malondialdehyde (MDA) production, leading to browning. Concurrently, browning-prone tissues frequently display suppression of key antioxidant systems such as the Ascorbate-Glutathione ASA-GSH cycle, which could diminish cellular redox buffering capacity and consequently lead to more severe browning [2, 15].

While sharing a core mechanism, interspecific variations exist concerning the identity of key phenolic substrates and the relative contribution of PPO activity versus phenolic content to browning. PPO substrates vary notably across species. Browning in eggplant is mainly caused by the oxidation of chlorogenic acid (CGA), which represents the most abundant phenolic compound in this horticultural species [16–18]. In contrast, apple possesses a more complex substrate pool, where proanthocyanidins, (+)-catechin, (−)-epicatechin, and phloridzin show stronger correlations with browning severity than CGA alone [19]. Intriguingly, CGA may serve as an antibrowning agent in apple, which represents a function opposed to its probrowning role in eggplant: *Md*MYB93, which activates CGA biosynthesis via *Md*HCT6, concurrently suppresses browning. This suggests that accumulated CGA may act as a potent antioxidant, scavenging ROS and potentially inhibiting PPO, thus linking enhanced CGA synthesis to reduced browning [20]. Furthermore, (−)-epicatechin is the primary substrate of PPO in litchi [14], while walnut PPOs exhibit high affinity for catechol, CGA, and epicatechin [10]. Moreover, the key factors influencing browning severity also vary across species. In apples, PPO activity serves as a key determinant, as the suppression of *PPO*s effectively prevents browning (e.g. Arctic® Apple). In eggplant, there seems to be a lack of consistency. Studies have shown that browning in fresh fruit depends on both PPO activity and phenolic contents, whereas total phenolic content emerges as the major limiting factor after storage [21]. Some other research suggests no significant correlation between total phenolics or chlorogenic acid contents and fruit browning, though PPO activity was moderately correlated with browning intensity [16]. These discrepancies may be attributed to several factors, including genetic differences, postharvest treatments, and quantification methods.

### Tyrosinase

TYRs (EC 1.14.18.1), members of the PPO superfamily, also play a critical role in the process of melanogenesis in fungi, animals, and some specific plants. The process mediated by TYRs involves two key steps: the hydroxylation of monophenols (e.g. *L*-tyrosine) to *o*-diphenols (e.g. *L*-DOPA), followed by the oxidation of these *o*-diphenols to *o*-quinones (Fig. 1) [6]. Subsequently, the resulting *o*-quinones polymerize non-enzymatically to form brown pigments (Fig. 1). This ability to initiate the browning cascade from monophenolic precursors distinguishes TYRs from COs.

Despite the prominent role of TYRs in plant browning documented in various literature, the existence and functional relevance of true tyrosinase enzymes in horticultural crops remain insufficiently understood [22]. Studies on recombinant PPOs from grape and apricot indicate that certain plant PPOs exhibit a low but detectable level of TYR activity alongside their predominant CO function [23, 24]. This expands the known functional diversity of plant PPOs, which includes isoforms that act primarily as COs, others that exhibit TYR activity, and some with dual catalytic capabilities. Notably, isoforms dedicated exclusively to TYR function appear rare in plants. Evolutionarily, this pattern may correspond with diversification in physiological needs across species: TYR activity is crucial for melanin synthesis in animals and fungi, whereas in plants, selective pressure might have favored the one-step CO activity for rapid defense responses. The minute TYR activity detected in some plants may represent a trait that has not been lost completely during evolution or a subsidiary metabolic capacity due to structural similarity in substrates, which indeed requires further exploration.

Given the critical role of TYRs in browning and the subsequent impact on postharvest losses in the food industry, the development of novel, safe, and effective TYR inhibitors has become a main goal in food postharvest management research. Most inhibitor screening still relies on the mushroom TYR model, through which several natural and synthetic compounds have shown promise. [25–29]. For instance, ascorbic acid (AA), a well-known antioxidant, inhibited mushroom TYR at high concentrations, and its antibrowning mechanism involves copper chelation at the active site, suppression of PPO and POD activities, and enhancement of ascorbate peroxidase (APX) activity, collectively inhibiting phenolic oxidation [28]. Other natural TYR inhibitors include esculetin, piceid, and tannins from longan shells [26, 27, 29]. To overcome limitations in the potency or stability of natural inhibitors, various synthetic analogs have been developed [25, 30, 31], and some have proven effective in controlling fruit browning. One example, *N*-Caffeoyl cysteine methyl ester (3o), demonstrates strong antioxidant and TYR inhibitory activity, which effectively reduced enzymatic browning in sliced apples and apple juice [25]. Despite these advances, the development of novel TYR inhibitors remains a crucial and ongoing challenge in food and biomedical sciences to effectively control enzymatic browning. It is also important to note that the mushroom TYR model has limitations for predicting efficacy in plants, owing to the phylogenetic distance between plants and fungi. This further highlights the need for future efforts to prioritize a plant–TYR-based verification model, as well as to characterize and inhibit the CO function central to browning in horticultural crops, even as the search for TYR inhibitors continues to be relevant.

### Laccase

LACs (EC 1.10.3.2), members of the multicopper oxidase family, are widely distributed across plants, fungi, and bacteria [32]. Though sharing overlapping enzymatic functions, LACs and PPOs represent phylogenetically distinct classes of enzymes with independent evolutionary origins in plants [6]. LACs oxidize a broad range of phenolic substrates, including monolignols, flavonoids, and aromatic amines, using molecular oxygen as an electron acceptor (Fig. 1). This oxidation generates highly reactive semiquinone radicals as key intermediates. Due to their instability, these radicals undergo rapid non-enzymatic reactions, including radical coupling, further oxidation, and cross-linking with other phenols, proteins, or polysaccharides. The subsequent polymerization of these complexes leads to browning in fruits.

Research on LAC-mediated fruit browning varies significantly in depth and focus across species, with litchi (*Litchi chinensis*) being the most thoroughly studied species. Pericarp browning in litchi is driven primarily by three key LACs: *Le*LAC7, *Le*LAC14–3, and *Le*LAC14–4 [33, 34]. Among these, *Le*LAC14–4 has received the most scientific attention. It was previously identified as an anthocyanin-degrading enzyme (ADE/LAC), which is responsible for the degradation of anthocyanins and the consequent loss of the red color in litchi pericarp during postharvest storage. The molecular role of *LeLAC14–4* in browning was validated via transgenic means: Its overexpression complemented the *transparent-testa* phenotype and restored the dark brown seed coat in the Arabidopsis mutant lacking *AtLAC15* [35], while the knockout of this gene attenuated browning in litchi [36]. *Le*LAC7, sharing a similar subcellular localization with *Le*LAC14–4*,* showed postharvest upregulation as well. It can oxidize (−)-epicatechin and coniferyl alcohol, suggesting a role of proanthocyanidin and lignin polymerization in litchi pericarp browning [32, 33]. Later work demonstrated that *Le*LAC14–3 is highly expressed in litchi pericarps and plays a critical role in the postharvest browning of litchi as well [32]. A few LACs have been studied in species other than litchi, for example, *DlLAC14–4*, an orthologue in longan (*Dimocarpus longan*), catalyzes procyanidin polymerization to induce pericarp browning [37]. The gene of an apple LAC, sharing 99% sequence homology with Arabidopsis and litchi LACs, was cloned and functionally characterized, showing increased activity in untreated fruit during storage and suppression by antibrowning agents (e.g. DPA, 1-MCP) [38]. In eggplant, the *LAC* gene family has been identified and bioinformatically characterized with predicted functions in some critical physiological processes (e.g. flavonoid biosynthesis) [39]. However, direct evidence linking specific LACs to browning is still lacking, which represents an underexplored but significant area for in-depth future research.

### Peroxidase

PODs (EC 1.11.1.7), members of the oxidoreductase family, catalyze the oxidation of a wide range of phenolic compounds in the presence of hydrogen peroxide (H₂O₂), leading to quinone radical formation and subsequent polymerization into insoluble brown pigments [8] (Fig. 1). Current evidence indicates that POD plays a context-dependent dual role in enzymatic browning, acting either as a promoter or suppressor depending on the physiological condition, tissue type, species, and temperature. In a comparative study of browning severity and PPO/POD activities across 14 apple cultivars, POD activity increased significantly across all cultivars between T1 (1 h postcut) and T2 (24 h postcut). This increase even exceeds that of PPO, suggesting a more prominent role of POD in the later stages (24 h) of postcutting browning. The regulatory role of POD is further interconnected with PPO activity, phenolic profile complexity, and potentially other oxidative enzymes like indole-3-acetic acid oxidase (IAAox) [8]. Additionally, in water caltrop (*Trapa taiwanensis* Nakai) pericarp, increased activity of POD within the 30–50°C range accelerated phenolic degradation and enhanced discoloration, directly implicating POD in heat-induced enzymatic browning [40].

Conversely, POD can serve as a key ROS-scavenging enzyme, potentially mitigating oxidative stress-associated browning in some plant species. For instance, slow-browning apples showed higher POD activity than fast-browning apples, and that POD correlated negatively with browning index. This protective role can be potentially attributed to synergistic interactions among POD, superoxide dismutase (SOD), and ascorbic acid in maintaining redox homeostasis, thereby delaying browning [41]. Studies also showed that cinnamic acid treatments, which increased POD activity, reduced browning in fresh-cut taro. Here, POD is interpreted primarily as an antioxidant, protecting tissues from oxidative damage that may potentially lead to browning [42]. A similar pattern was observed in fresh-cut lotus roots treated with 24-epibrassinolide [43]. Interestingly, increased POD activity in *StODO1*-overexpressing potato is associated with reduced ROS levels but more severe browning [44], highlighting that a disconnection between POD’s roles in ROS scavenging and browning promotion in this genetically modified species. Overall, browning outcomes in various fruit species depend on a delicate balance among the relative activities of POD and PPO, the availability of H₂O₂ and phenolic substrates, and the specific genetic background of the plant, which makes POD a critical but complex factor in postharvest physiology and a potential target for browning control that require deep mechanistic interpretation and precise modulation.

### Enzymes of the flavonoid biosynthesis pathway

Research on the mechanisms of postharvest fruit browning has extended beyond the traditional view, with PPO and POD being the main players. Accumulating evidence indicates that the degree of browning does not always correlate with the activity of these two enzymes [45–47], suggesting the presence of alternative browning mechanisms that operate independently of phenolic oxidation. This perspective is supported by recent work concerning the color shift in eggplant (*Solanum melongena* L.) fruits during ripening. Eggplant fruit accumulates purple anthocyanins early in development and then switches to a different set of pigments (yellow-brown) later. The color shift is mediated through the expression modulation of both late biosynthetic genes (LBGs, reducing anthocyanins) and early biosynthetic genes (EBGs, accumulating naringenin chalcone and flavonols) by a complex regulatory network involving TFs and miRNAs. The coordination between LBGs, including dihydroflavonol 4-reductase (*DFR*), anthocyanidin synthase (*ANS*)*,* and multiple glycosyltransferases (*GT*s), and EBGs, phenylalanine ammonia-lyase (*PAL*), cinnamate 4-hydroxylase (*C4H*), 4-coumaroyl-CoA ligase (*4CL*), and chalcone synthase (*CHS*), as well as flavonol synthase (*FLS*), redirects flux toward naringenin chalcone and flavonol production, ultimately resulting in the characteristic yellow-brown color in ripe eggplant fruits [4], indicating the role of the flavonoid pathway in color development.

As the entry point enzyme in the phenylpropanoid pathway, PAL (EC 4.3.1.24) initiates the biosynthesis of a diverse array of phenolic compounds (e.g. flavonoids, anthocyanins) [3], thus linking specific cues to enhanced phenolic metabolism and subsequent discoloration. PAL activation promotes browning by either synthesizing phenolic substrates for oxidative enzymes or producing flavonoids with yellow or brown hues independently of PPO activity [3]. Accumulating evidence from various species supports the critical role of PAL in regulating browning. Specifically, cut-surface yellowing in Chinese water chestnut (*Eleocharis tuberosa*) was inhibited by exogenous ferulic acid, which corresponds with decreased PAL activity and subsequent reduction in eriodictyol and naringenin [48]. More convincingly, PAL activity was completely repressed in acetic acid-treated lettuce with no obvious browning, whereas PPO activity was increased, which demonstrates a lack of correlation between PPO and browning in this species [46]. A similar observation was made in potatoes as well, where browning is partially correlated to PAL, but not to PPO or POD [47]. These findings collectively place PAL as a central node in the browning regulatory network, capable of driving color change through mechanisms that extend beyond conventional enzymatic oxidation pathways.

## Molecular regulation of enzymatic browning

The expression levels and activities of browning-related genes represent a key determinant in the regulation of enzymatic browning, which is subject to complex molecular regulation at multiple levels. This multilayered control includes the action of TFs that modulate the initiation of gene transcription, miRNAs that regulate gene expression by inhibiting the translation of mRNA transcripts after they are made, and epigenetic modifications, which can heritably silence genes without altering the DNA sequence. The integration of these sophisticated regulatory levels controlling the key genes specifies the ultimate and diverse browning phenotypes in various species in a particular context.

### Transcriptional regulation via transcription factors

**Figure 2:**
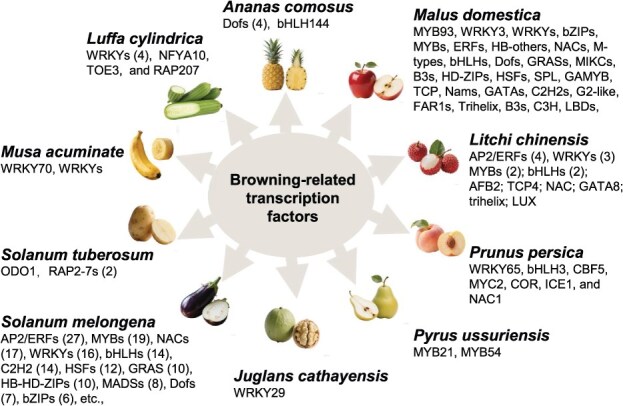
TFs implicated in browning regulation in various fruits and vegetables. The quantity of transcription factors within a given family is denoted within parentheses.

**Figure 3:**
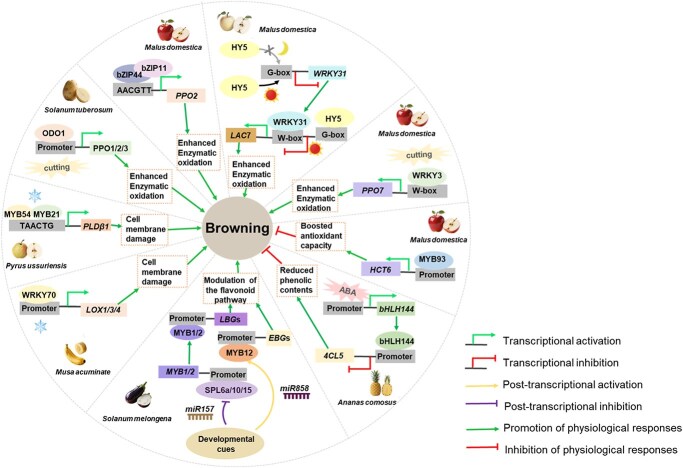
Mechanistic insights into browning regulation by TFs in well-studied fruit species.

Acting as molecular switches in response to diverse internal and external stimuli, TFs orchestrate the spatiotemporal expression of specific gene sets, thereby controlling fundamental physiological processes, including enzymatic browning. Recent advancements in multi-omics technologies have facilitated the systematic identification of TFs associated with browning in diverse fruits and vegetables, including pineapple (*Ananas comosus* L.) [49], walnut (*Juglans cathayensis* Dode) [50], sponge gourd (*Luffa cylindrica*) [51, 52], apple (*Malus domestica* Borkh.) [20, 41, 53–57], eggplant (*S. melongena* L.) [4, 18], peach (*Prunus persica*) [58–61], banana (*Musa acuminate*) [62, 63], and litchi (*L. chinensis* Sonn.) [64, 65] (Fig. 2 and Table S2). Though numerous candidate TFs have been identified through omics-based approaches, the mechanistic insights into their roles in regulating browning remain limited. To date, only a few TFs have been thoroughly investigated and validated (Fig. 3). Current evidences suggest that TFs act as central regulatory hubs, converting postharvest environmental signals into precise transcriptional programs that activate downstream biochemical pathways. This integrated framework is characterized by regulation convergence, species adaptation, and functional diversification, collectively shaping the complexity of browning responses across horticultural crops.

TF-mediated browning regulation converges on a limited set of core biochemical pathways, including membrane lipid degradation, enzymatic oxidation, and phenolic metabolism. Current evidence indicates that membrane lipid degradation triggered by cold stress is primarily regulated by WRKY [62, 63] and MYBs [66]. As a key process that ultimately determines the browning phenotype in fruits, oxidation facilitated by oxidative enzymes (e.g. PPOs and LACs) is strongly activated by TFs from the MYB [44], WRKY [53, 56], and bZIP [67] families. Furthermore, phenolic biosynthesis is regulated by MYB or bHLH TFs in different species, resulting in diverse physiological outcomes, including enhanced antioxidant capacity [20], restricted supply of browning precursors [68], or the accumulation of yellow-brown flavonoid pathway intermediates [4].

TF-mediated browning regulation in plants is characterized by significant functional versatility and specialization within and across TF families. Members of the same TF family may possess distinct functions in different species. Take MYBs, e.g. potato *St*ODO1 directly activates *PPO* genes to promote enzymatic browning upon cutting [44], whereas in cold-stored ‘Nanguo’ pears, *Pu*MYB21 and *Pu*MYB54 jointly promote membrane degradation by activating a phospholipase D gene *PuPLDβ1*, thereby facilitating phenolic–PPO contact and browning [66]. Conversely, *Md*MYB93 in apple regulates CGA biosynthesis, exerting a protective, antibrowning function [20]. In eggplant, different MYBs specifically regulate anthocyanin and chalcone accumulation related to color shift during ripening [4]. Similarly, the WRKY family members also function in species-specific and context-dependent manner. In banana, cold-induced *Ma*WRKY70 promotes chilling injury by activating lipoxygenase (*LOX*) genes involved in membrane degradation [62]. In apple, wound-induced *Md*WRKY3 activates *PPO* to drive flesh browning [56], while darkness-induced *Md*WRKY31 activates *LAC*, leading to peel browning [53]. Furthermore, though the bZIP family members are consistently involved in the upregulation of genes encoding oxidative enzymes to promote browning, their targets and mechanistic details differ, and the underlying mechanisms vary across species [53, 67]. Functional diversification within TF families extends to a convergent evolutionary strategy, where different TF families in different species regulate similar physiological processes, such as membrane lipid degradation, which is regulated by WRKY in banana [62] but MYB in pear [66].

Within a given species, multiple TFs may be involved in regulating different physiological processes to collectively influence the outcome of browning. As in apples, a sophisticated TF network integrates diverse environmental signals and coordinates to fine-tune browning. Mechanical injury could activate *Md*bZIP44 and *Md*WRKY3, which both drive flesh browning by directly inducing the expression of different *PPO* isoforms, *MdPPO2* and *MdPPO7*, respectively [67]. Light/dark conditions govern browning via a protein module involving *Md*WRKY31 and *Md*HY5, a TF from the bZIP family. *Md*WRKY31 enhances ‘Ruixue’ apple flesh browning under darkness via the upregulation of *MdLAC7*, a process that is suppressed by light through the photo-repressive *Md*HY5 [53]. Beyond these direct browning pathways, *Md*MYB93 operates in an antioxidant module by activating CGA biosynthesis, thereby promoting postharvest resilience [20]. The existence of parallel regulatory pathways even within a single species highlights a profound level of biological complexity, suggesting that enzymatic browning is governed by a dynamic, context-dependent interplay of multiple factors rather than a simple linear cascade. This complexity calls for deeper investigation into the hierarchies and crosstalk among them that ultimately define phenotypic outcomes.

### miRNA-mediated post-transcriptional regulation

**Figure 4:**
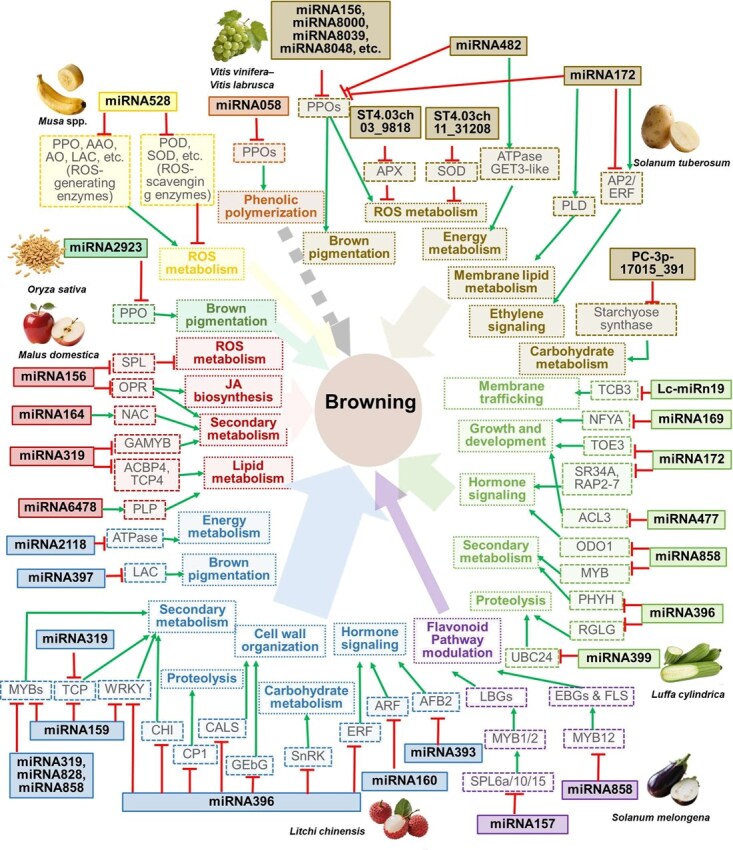
Regulatory network of miRNAs implicated in browning regulation in fruit and vegetable species. miRNAs, their putative targets, and the regulated physiological processes are shown.

Research on miRNA-mediated regulation of fruit and vegetable browning has increasingly focused on the systematic identification and functional validation of miRNAs and their target genes. Extensive studies across multiple species, including banana (*Musa* spp.), sponge gourd (*L. cylindrica*), apple (*M. domestica*), and litchi (*L. chinensis*), have led to the establishment of a tentative miRNA regulatory network governing browning (Fig. 4 and Table S3) [51, 65, 69–71].

Accumulating evidence implicates miRNAs in enzymatic browning regulation by directly targeting key browning genes. For instance, eight conserved miRNAs (e.g. stu-miR482e-5p, stu-miR8039, stu-miR172b-5p) in cut potato (*Solanum tuberosum*) tubers were predicted to target three *PPO* genes directly. Notably, these miRNAs exhibit low expression in tuber tissues, suggesting that inadequate repression of *PPO*s by miRNAs contributes to enzymatic browning in potato tubers [72]. This is consistent with the regulatory role of miRNA058 shown in the network, which also targets PPOs and influences phenolic polymerization [73]. In bananas, cold stress downregulated miRNA528, which led to *PPO* upregulation and increased ROS levels, promoting peel browning [69]. Similarly, in rice, miRNA2923a was identified to target *PPO*, which not only prevents browning but also protects plants against pathogens and environmental stresses [74]. In addition to targeting *PPO*s, miRNAs are also involved in the expression modulation of genes responsible for the production of flavonoid-pathway intermediates associated with browning. In eggplant, miR157 and miR858 act synergistically to regulate the shift from purple anthocyanin-based pigmentation in young fruit to yellow naringenin chalcone accumulation in ripe fruit through a coordinated transcriptional and post-transcriptional regulation of both transcription factors and flavonoid biosynthetic genes [4]. Beyond direct suppression of browning-related genes, miRNAs coordinate interconnected physiological processes, including carbohydrate metabolism, redox homeostasis, hormone signaling, and energy metabolism, to collectively modulate enzymatic browning in diverse fruits and vegetables (Fig. 4) [51, 65, 69–71].

Although different plant species often utilize a distinct set of miRNAs to regulate browning, functional consistency is observed across species. Based on the established network, miRNA858, miRNA319, and miRNA396 converge on flavonoid and phenylpropanoid pathways, while miRNA396 and miRNA172 intersect with proteolytic and hormone-mediated processes, highlighting the multilayered and species-specific nature of miRNA regulation in browning [4, 51, 55, 65, 71]. Certain miRNAs target conserved functional pathways through species-specific gene targeting. For example, miRNA396 in sponge gourd targets *RGLG* (E3 ubiquitin-protein ligase RGLG2-like protein) and *PHYH* (phytanoyl-CoA dioxygenase) genes, which consequently regulate proteolysis and secondary metabolism, respectively [51]. In litchi, miRNA396 targets a different set of genes (*CP1* for proteolysis; *WRKY* for secondary metabolism) and exhibits functional diversification by additionally suppressing genes involved in regulating cell wall organization, carbohydrate metabolism, and hormone signaling [65]. Similarly, the role of miRNA172 in regulating browning through hormone signaling and processing is demonstrated in both potatoes [72, 75] and sponge gourds [51], with their potential targets being different in these two species. Furthermore, key regulatory hubs or processes are frequently targeted by multiple miRNAs across diverse species. For example, MYBs are targeted by miRNA 319 in apple and litchi [55, 65] while by miRNA858 in litchi [64], sponge gourd [51], and eggplant [4]. This pattern reflects a conserved but adaptable miRNA-mediated regulatory strategy, where evolutionary adaptation and diversification enables fine-tuned control of browning in different species.

### Epigenetic modifications in browning regulation

Research increasingly suggests that epigenetic modifications play a critical role in regulating fruit and vegetable browning (Table S4). Among different types of epigenetic modifications, DNA methylation emerges as a critical regulatory module governing enzymatic browning. This link between DNA methylation and regulated browning was first established in peach (*P. persica*), where transcriptomic and methylomic analyses revealed a strong positive correlation between global DNA methylation levels and internal browning induced by storage at ≤12°C [58, 59]. Subsequent multi-omics analyses comparing two natural bud mutation varieties of ‘Fuji’ apple further demonstrated that browning severity correlated positively with both global cytosine methylation (5mC%) and the expression of key methyltransferases (*MdCMT3*, *MdCMT3c*), but negatively with demethylase (*MdROS1*, *MdDME*) [76]. Additional supporting evidence shows that reducing global DNA methylation mitigates peel browning in banana [77], confirming the conserved role of DNA methylation in regulating browning across species.

DNA methylation regulates fruit browning by targeting key structural genes involved in the process. In peach, methylomic analyses revealed that differential methylation influenced the expression of *PpPPO1/2* (pigment formation), lipoxygenase *PpLox1* (membrane stability), and superoxide dismutase *PpSOD1/2* (oxidative stress), collectively promoting brown pigment accumulation during cold storage [59]. Melatonin application alleviates browning by inducing hypermethylation in the CpG islands of *PpPPO* and *PpPOD* promoters, suppressing their transcription and reducing enzymatic browning, while causing hypomethylation of *PpPAL*, enhancing phenylpropanoid pathway flux, and further mitigating browning in cold-stored fruit [78]. Beyond directly regulating key browning genes, DNA methylation indirectly influences browning by modulating transcription factors, competing metabolic pathways, and hormone-related genes, enabling cross-pathway coordination. In apple, hypermethylation of NAC1, a key transcription factor that chaperones catalase and maintains ROS homeostasis, suppresses its expression and accelerates browning [76]. The role of methylated NCA1 was further supported in peach: Methyl jasmonate (MeJA) reduced internal browning under cold storage by demethylating the NCA1 promoter and restoring its expression [61]. Additional studies linked browning severity in cold-stored peaches to methylation levels in genes related to ethylene biosynthesis, cell wall metabolism, and ROS homeostasis, with region-specific methylation playing distinct roles [59, 60].

In addition to DNA methylation, evidence also suggests a potential role of RNA methylation (e.g. m6A) and histone modification in browning regulation. As indicated by a transcriptome-wide m6A-seq analysis of nitrogen-treated potatoes, browning severity was correlated with the expression of genes encoding components of miRNA machinery, such as metallothionein-like protein type 2A (an m6A writer) and ethanolamine-phosphate cytidylyltransferase-like (an m6A reader) [79]. In grapes, VlSRT1, a histone deacetylase, is responsible for reducing H3 acetylation at promoters of ethylene and chlorophyll catabolism genes *VlACS5* (1-aminocyclopropane-1-carboxylic acid synthase) and *VlPAO1* (Pheophorbide a oxygenase), thereby suppressing their expression and delaying rachis browning [80]. In comparison with DNA methylation, research on the role of RNA modifications and histone modifications in browning regulation is still at an early stage, representing a promising frontier for future investigation. Additionally, to fully elucidate the ‘big picture’ of epigenetic regulation in browning, more systematic and in-depth functional studies are required, along with exploration of the crosstalk between various types of epigenetic modifications.

## Hormonal regulation and practical applications

**Figure 5:**
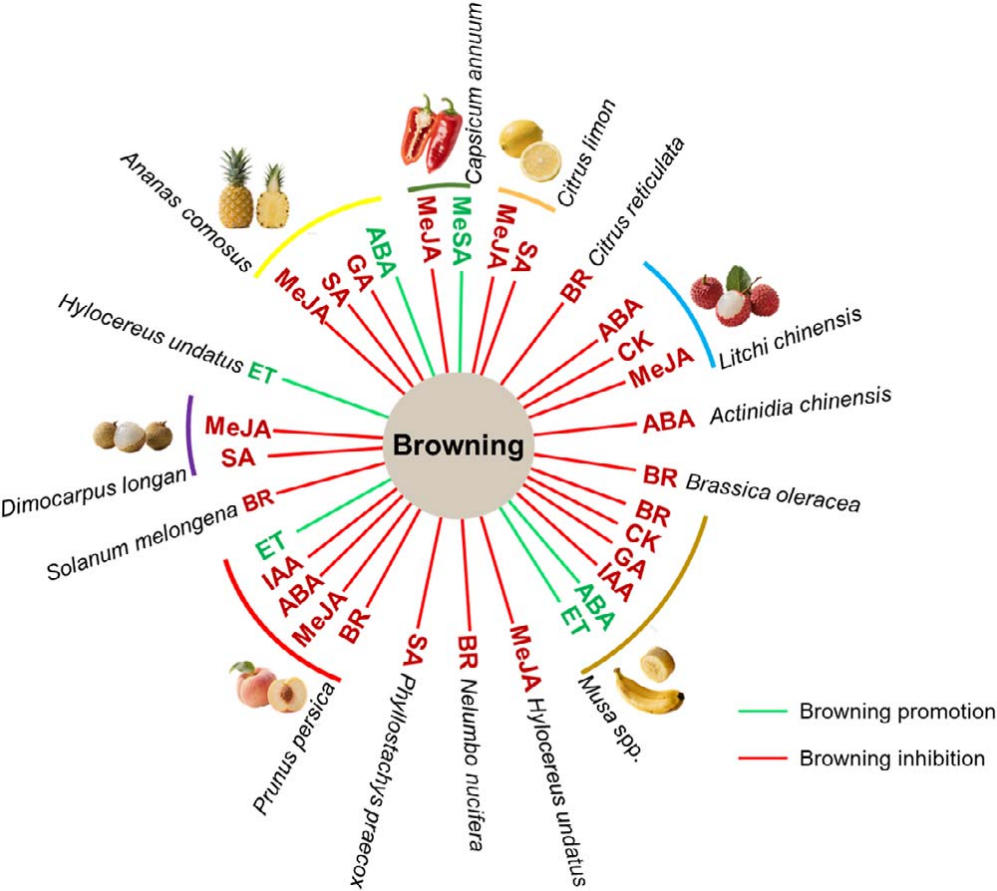
The effects of phytohormone applications on browning regulation in diverse species.

Fruit browning is a complex physiological and biochemical process with phytohormones serving as its key regulators, and the specific role of each phytohormone in modulating fruit and vegetable browning has been extensively documented in numerous studies (Fig. 5 and Table S5). Exogenous application of plant hormones provides an effective strategy to mitigate browning in vegetables and fruits, while avoiding the potential risks of genetic modification.

### Auxin

Indole-3-acetic acid (IAA), the primary auxin phytohormone, exhibits context-dependent effects on fruit browning. Exogenous IAA application suppressed peel browning in banana (*Musa acuminata*), and this effect was counteracted by abscisic acid [81]. Similarly, in peach (*P. persica*), IAA treatment reduced chilling-induced flesh browning through the upregulation of auxin biosynthetic genes (*PpMES13*, *PpMES17*) and auxin-responsive genes (*PpSAURs*), coupled with suppression of degradation genes (*PpDAO*, *PpGH3*), leading to enhanced IAA accumulation and browning alleviation [82]. Interestingly, Auxin may promote browning under specific contexts. In litchi, miRNAs that inhibit auxin and ethylene signaling were highly expressed in fruits with alleviated browning, providing indirect evidence that auxin signaling contributes to browning promotion [65]. Further support comes from cold-stored pepper (*Capsicum annuum* L.), where a chilling-sensitive cultivar exhibited severe seed browning accompanied by enhanced IAA accumulation and elevated ROS [83].

### Ethylene

Ethylene (ET) also plays context-dependent dual roles in fruit browning, exhibiting both promotive and alleviating effects depending on species, treatment, and storage conditions. Exogenous ET exacerbated brown spot formation on banana peels, which can be reversed by the DNA demethylating agent, azacytidine [77]. Inhibition of ethylene action or perception also reduces browning. Treatment with 1-methylcyclopropene (1-MCP), an ethylene inhibitor, alleviated peel browning in cold-stored ‘Nanguo’ pear (*Pyrus ussuriensis*), possibly by enhancing energy status, reducing oxidative stress, and maintaining membrane integrity [84], and similarly reduced enzymatic browning in nectarine [85]. Conversely, ethylene can delay the onset of browning under specific conditions in different species. In peach, ethylene deficiency under chilling stress promoted internal browning by upregulating browning-related enzymes, disrupting membrane stability, and reducing ROS homeostasis [59]. In apples, inhibited ethylene production by cold storage or treatment with 1-MCP also led to progressive flesh browning development during the following controlled ambient storage [86]. More directly, Exogenous ethylene alleviated peel browning in ‘Huangguan’ Pear by enhancing the activity of antioxidant enzymes (e.g. CAT, APX, SOD), suppressing PPO activity, reducing electrolyte leakage, and lowering respiration rate [87].

### Cytokinins

Cytokinins (CKs), a principal class of phytohormones regulating cell division, can also delay senescence and mitigate postharvest browning in fruits. Exogenous application of natural cytokinins or synthetic analogs such as 6-benzylaminopurine (BAP) and forchlorfenuron (CPPU) effectively reduces browning across fruit species. In litchi, CK treatment led to a thicker pericarp, reduced water loss, and therefore decreased browning susceptibility [88]; application of BAP also inhibited litchi postharvest decay, potentially by inhibiting pathogenic fungi, ROS accumulation, and PPO activity, while enhancing anthocyanin production and ROS-responsive enzyme activities [89]. In bananas, CPPU delayed ripening and brown spot formation by upregulating cytokinin synthesis genes (*MaIPT1/12*) while downregulating cytokinin degradation genes (*MaCKX1/12*), leading to elevated endogenous CK levels. Along with concurrent suppression of chlorophyll degradation genes (e.g. *MaPAO1*, *MaCLH1*), browning was alleviated [90,91].

### Abscisic acid

Although decreased abscisic acid (ABA) levels have been linked to browning inhibition [64,92,93] in some species, more evidence supports its antibrowning role. The underlying mechanisms are complex and vary by fruit type and application dosage. In peach, ABA alleviates chilling injury-related browning by enhancing cold tolerance through sucrose preservation, which acts as a cryoprotectant and osmotic regulator [94]. In kiwi fruit (*Actinidia chinensis*), ABA exhibits dose-dependent effects: low doses reduced pulp browning by enhancing peel lignification and antioxidant capacity, whereas high doses promoted excess lignification in both pulp and peel, accelerating browning [95]. In pineapple, ABA application significantly reduces internal browning by inhibiting key enzymes (PAL, PPO, POD) and maintaining ascorbic acid (antioxidant) levels [96]. Mechanistically, ABA suppresses phenolic-mediated browning by upregulating TF *AcbHLH144*, which represses the phenolic biosynthesis gene *Ac4CL5*, reducing phenolic accumulation [68].

### Gibberellic acid

Gibberellin (GA) also exhibits species-specific and context-dependent roles in browning regulation, with evidence supporting its contrasting effects in diverse fruit species. In pineapples, GA application enhanced flesh browning in both chilling-tolerant and sensitive cultivars [97]. This effect is likely due to GA-induced enhancement of PPO expression and activity [97,98], as well as modulation of spermidine (a browning alleviator) accumulation [99]. In contrast, postharvest GA treatment retards ripening and reduces peel browning in bananas [100], an effect enhanced when combined with CPPU [91,101]. Supporting its anti-browning role in other species, elevated GA levels were associated with arrested ripening and reduced browning in AZA-treated strawberries [93], and exogenous GA alleviated browning in litchi by inhibiting anthocyanin accumulation [102].

### Jasmonate, salicylate, and their methylated forms

MeJA and salicylic acid (SA) are signaling molecules that effectively mitigate postharvest fruit browning. Substantial evidence supports MeJA’s efficacy in reducing browning in diverse fruits, including litchi, peach, prune (*Prunus domestica*), dragon fruit (*Hylocereus undatus*), and longan (*D. longan*), and its predicted antibrowning mechanism involves the modulation of browning-related enzyme activity, energy metabolism, membrane stability, and oxidative hemostasis [103–111]. Similarly, SA treatment reduces pericarp browning in longan by enhancing antioxidant capacity and inhibiting membrane lipid degradation [112], and its role in browning alleviation is also evident in bamboo shoots (*Phyllostachys praecox*) [113]. In contrast, Methyl salicylate (MeSA) exhibits species-dependent effects on browning: it significantly reduced flesh browning in dragon fruit by suppressing respiration, ethylene production, electrolyte leakage, and lipid peroxidation [114], but promotes seed browning in peppers by reducing antioxidant content and impairing ROS scavenging [115].

### Brassinosteroids

Brassinosteroids (BRs) are essential steroid hormones regulating diverse physiological processes in plants, and their effects on browning regulation have been documented in diverse fruits and vegetables, such as banana, peach, guava, lotus, and kale [43,116–124]. The antibrowning role of BRs, particularly under cold storage conditions, has been exclusively demonstrated in all related studies. Evidence suggests that BRs mitigate chilling injury and browning, possibly through the integration of membrane integrity preservation, antioxidant system enhancement, and strategic phenolic metabolism regulation.

Phytohormone-mediated browning regulation operates through a complex and integrated network that converges on a few key physiological hubs, including oxidative homeostasis, secondary metabolism, membrane integrity, energy status, and cellular signaling. The function of any given hormone, however, is highly variable and sometimes contradictory across different plant species, with the ultimate browning phenotype depending on species, tissue, developmental stage, and postharvest conditions. This intricate network allows for fine-tuned adaptation of different species in response to various environmental stimuli and also explains why the practical application of exogenous hormones requires precise, species-specific optimization to effectively control browning.

## Browning control strategies

Due to the agronomic and economic challenges posed by enzymatic browning, efforts have been directed toward developing strategies to manage this process, primarily by intervening in postharvest physiological and biochemical pathways [2,125]. These strategies can be broadly categorized into chemical and physical methods. Chemical approaches employ exogenous compounds to directly interrupt the browning pathways. These agents act through diverse mechanisms. Chelating agents (e.g. EDTA, citrate) and acidulants (e.g. SO₂, ascorbic acid, chlorine dioxide) inactivate PPO directly by sequestering its copper cofactor and reducing pH, respectively; phytohormones and signaling molecules (e.g. ethylene, 1-MCP) orchestrate postharvest browning through a multifaceted regulatory network. They precisely modulate this process by targeting key components, including enzyme activity, substrate availability, membrane integrity, and the cellular redox state; protective metabolites (e.g. GABA, polyamines) and antioxidants (e.g. α-lipoic acid, propyl gallate) enhance stress tolerance, or cellular energy status; thereby alleviating browning [1,2,125–127]. In parallel, there has been a growing interest in the use of plant natural products in enzymatic browning intervention. These compounds are of particular interest due to their natural origin, and they often exert effects through a combination of the mechanisms described above [125,128]. Physical treatments, in contrast, delay browning by modifying the postharvest environment. Key methods include cooling to maintain cellular integrity, controlled atmospheres to reduce respiration, UV irradiation for direct PPO suppression, and the application of edible coatings (e.g. chitosan) that form a semipermeable barrier to alter internal gas composition and oxidative state [2,126,127]. Despite the development of diverse strategies, postharvest losses associated with enzymatic browning remain unresolved, largely due to the significant inherent limitations of current methods. The efficacy of many approaches is highly species- and context-dependent, often requiring extensive optimization but lacking broad applicability. Some approaches may only provide transient protection due to compound degradation or poor uptake in plant tissues [126]. More critically, certain synthetic agents are related to risks of toxicity/health issues or raise environmental concerns regarding their persistence and biocompatibility [128,129]. Additionally, the current intervention approaches face considerable challenges in the economic aspect, as they may entail substantial ongoing costs for chemical reagents, as well as significant initial investment and maintenance expenses for a specialized facility.

To address enzymatic browning beyond postharvest browning prevention, an integrated breeding strategy combining conventional and molecular approaches offers a comprehensive solution. Systematic germplasm screening and molecular marker-based analysis expand genetic diversity and identify new browning-tolerant accessions, while allele mining is useful for elucidating the underlying mechanisms [130–132]. These insights enable marker-assisted selection, introgression, and trait pyramiding to develop varieties with multiple browning-resistance loci. In parallel, molecular design provides precise interventions through: direct disruption of PPO genes via CRISPR-Cas9-mediated knockout; limiting phenolic substrate availability by editing core phenylpropanoid-pathway genes or enhancing vacuolar sequestration to spatially separate phenolics from PPO [133,134]; strengthening cellular antioxidant capacity through enhancing ascorbate-glutathione cycle enzymes or catalase, thereby improving redox homeostasis and quenching reactive quinones [135,136]; leveraging master transcriptional regulators to downregulate gene networks governing both phenolic biosynthesis and PPO expression; and expediting breeding cycles via marker-assisted selection using functionally validated alleles of browning-related genes, facilitating rapid pyramiding of resistance traits into elite germplasm. The systematic convergence of natural genetic resource exploitation and precision molecular engineering establishes a robust pipeline for developing next-generation crops with superior postharvest performance, extended shelf life, and enhanced commercial viability. However, this will require comprehensive deciphering and precise manipulation of the core regulatory network underlying browning across species, which represents the main focus of this review.

## Concluding remarks and future perspectives

**Figure 6:**
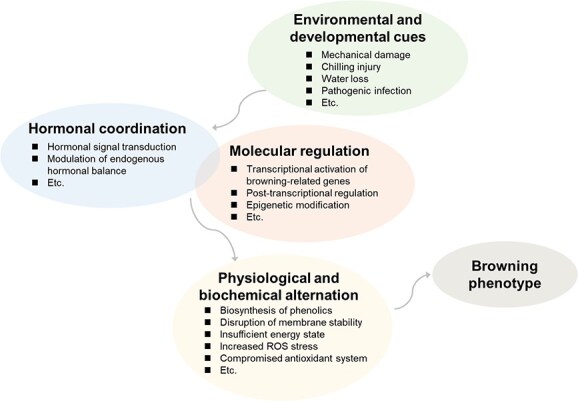
The dynamic regulatory network of browning in plants.

Fruit and vegetable browning is a sophisticated physiological process governed by a dynamic network that integrates multiple metabolic pathways across biological hierarchies (Fig. 6). Moving beyond a traditional focus on enzymatic players such as PPOs is essential to grasp its full complexity. A comprehensive mechanistic understanding of enzymatic browning requires elucidation as to how regulation operates within and across these levels, including the establishment of causal relationships and sequential orders, as this knowledge forms the foundation for developing effective control strategies. Current evidence indicates that browning regulation involves both conserved and species-specific mechanisms; this ‘unity and diversity’ likely arose from evolutionary adaptations to both biological and environmental triggers, a perspective that deserves further investigation. Understanding the evolutionary mechanisms behind the divergence and convergence of browning pathways and finding the key core regulatory mechanisms is crucial for translating knowledge into commercially viable and scalable browning-controlling strategies. However, significant research challenges persist due to the inherent complexity of browning. The intricate signaling and metabolic networks that drive browning are still not fully resolved. A critical gap lies in understanding how regulation is coordinated across molecular, cellular, physiological, and epigenetic levels. This challenge is also compounded by the multifactorial nature of environmental triggers and the poorly understood interactions among these triggers and/or with biological factors. Furthermore, translating mechanistic discoveries into practical applications proves to be uneasy.

The current limitations in the mechanistic understanding of fruit browning stem partially from methodological constraints that impede reliable cross-study comparison, data integration, and the establishment of clear regulatory relationships from large-scale datasets. At the biochemical level, the absence of standardized protocols for enzymatic assays (e.g. substrate concentration, pH, temperature, etc.) and a reliable verification system makes data comparison difficult and may even lead to contradictory conclusions across studies. Similarly, the assessment of browning severity relies on diverse, non-uniform indices, which further challenge the interpretation of results. Experimental setups are often insufficiently reported in published work or inconsistently controlled, resulting in low experimental reproducibility. Moreover, though multi-omics technologies have generated vast amounts of data, significant challenges remain in data management, establishing causal regulatory networks, and effectively linking omics findings to precise phenotypic outcomes.

To address these challenges, future research should adopt integrative and innovative strategies. A systems biology approach integrating multi-omics data across molecular, cellular, and organismal levels, followed by rigorous validation under physiologically relevant conditions, can be employed to decipher the complex regulatory networks driving browning and elucidating their interplay with diverse environmental stimuli. Building upon this foundation, mechanistic investigation of specific regulatory components should prioritize the following research areas including: validating the roles of specific transcription factors and their interaction in regulating browning; identifying the precise targets and characterizing the regulatory roles of miRNAs in the browning cascade, conducting comprehensive genome-wide profiling of dynamic epigenetic marks coupled with functional validation, and elucidating the interplay of diverse phytohormones in modulating browning. Moreover, robust comparative frameworks across diverse species should be established to distinguish conserved mechanisms from species-specific adaptations, providing a foundation for broadly applicable control strategies. Methodologically, promoting the standardization of experimental methods, improving the reporting of experimental conditions, and developing analytical methods that can integrate multi-omics data with genetic validation are crucial steps for advancing from fragmented observations to a mechanistic understanding. Ultimately, such integrated efforts are indispensable for bridging the gap between mechanistic insights and the development of effective postharvest solutions.

## Supplementary Material

Web_Material_uhag060

## Data Availability

There is no new data associated with this article.
